# Identification of three new *Alu* Yb subfamilies by source tracking of recently integrated *Alu* Yb elements

**DOI:** 10.1186/1759-8753-4-25

**Published:** 2013-11-12

**Authors:** Musaddeque Ahmed, Wen Li, Ping Liang

**Affiliations:** 1 Department of Biological Sciences, Brock University, St Catharines, Ontario L2S 3A1, Canada; 2 Institute of Reproductive and Stem Cell Engineering, Central South University, Changsha, Hunan, China

**Keywords:** Mobile element, *Alu*, evolution, *Alu* Yb8a1, *Alu* Yb10, *Alu* Yb11

## Abstract

**Background:**

*Alu* elements are the most abundant mobile elements in the human genome, with over 1 million copies and constituting more than 10% of the genome. The majority of these *Alu* elements were inserted into the primate genome 35 to 60 million years ago, but certain subfamilies of *Alu* elements are relatively very new and suspected to be still evolving. We attempted to trace the source/master copies of all human-specific members of the *Alu* Yb lineage using a computational approach by clustering similar Yb elements and constructing an evolutionary relation among the members of a cluster.

**Results:**

We discovered that one copy of Yb8 at 10p14 is the source of several active Yb8 copies, which retrotransposed to generate 712 copies or 54% of all human-specific Yb8 elements. We detected eight other Yb8 elements that had generated ten or more copies, potentially acting as 'stealth drivers’. One Yb8 element at 14q32.31 seemed to act as the source copy for all Yb9 elements tested, having producing 13 active Yb9 elements, and subsequently generated a total of 131 full-length copies. We identified and characterized three new subclasses of Yb elements: Yb8a1, Yb10 and Yb11. Their copy numbers in the reference genome are 75, 8 and 16. We analysed personal genome data from the 1000 Genome Project and detected an additional 6 Yb8a1, 3 Yb10 and 15 Yb11 copies outside the reference genome. Our analysis indicates that the Yb8a1 subfamily has a similar age to Yb9 (1.93 million years and 2.15 million years, respectively), while Yb10 and Yb11 evolved only 1.4 and 0.71 million years ago, suggesting a linear evolutionary path from Yb8a1 to Yb10 and then to Yb11. Our preliminary data indicate that members in Yb10 and Yb11 are mostly polymorphic, indicating their young age.

**Conclusions:**

Our findings suggest that the Yb lineage is still evolving with new subfamilies being formed. Due to their very young age and the high rate of being polymorphic, insertions from these young subfamilies are very useful genetic markers for studying human population genetics and migration patterns, and the trend for mobile element insertions in the human genome.

## Background

*Alu* elements are the most successful short interspersed elements (SINEs) in primate genomes. *Alu* elements have proliferated significantly throughout primate evolution and have expanded to more than 1 million copies in the human genome, constituting over 10% of the genome by mass [1,2]. The majority of these elements are suspected to have been inserted in the primate genome 35 to 60 million years ago, and since then the proliferation rate has reduced significantly by over 100 fold [3]. Thus, despite the large number of copies present in the human genome, only a small fraction of *Alu* elements are still active and capable of generating new copies [4-6]. The activity of *Alu* elements has generated different subfamilies of varying ages, each subfamily being defined and characterized by a set of diagnostic mutations [7]. Each subfamily is thought to have expanded when its master or source copy accumulated a mutation and then actively transposed to new locations at different rates and time periods of evolution [8,9].

The vast majority of the *Alu* elements currently found in the human genome were inserted before the divergence of humans and chimps, and thus are shared by all individuals of both species. The small fraction of *Alu* elements that have been recently inserted into the human genome are mostly restricted to several closely related young subfamilies, with the majority of these young elements being from the Ya5 and Yb8 *Alu* subfamilies [10,11]. Since almost all of these young *Alu* elements were inserted into the human genome after the human–chimp divergence, they are only found in humans. Some of these young active *Alu* elements have accumulated new mutations and have acted as source or master copies by generating subsets of elements that are identifiable as new subfamilies. Some of these subfamilies are so recent that they have members that are polymorphic for their presence or absence between individuals and/or populations [12-14]. The availability of a complete human reference genome and large quantities of individual genomic data from the 1000 Genome Project have facilitated the identification of these subfamilies and their level of polymorphism [15,16]. The homoplasy-free nature of *Alu* elements makes their polymorphic insertions very useful in phylogenetic studies, human population studies, forensics and DNA fingerprinting [9,17-20].

Our study specifically focuses on human-specific *Alu* elements from the Yb lineage, mainly because they are the second largest young family by the number of copies in the human genome, comprising 40% of all human-specific *Alu* elements with more than 30% of these copies being polymorphic between individuals and/or populations [15,16,21]. *Alu* Yb8 is the major subset of this family. Its high rate of being human-specific and polymorphic among humans and its involvement in human diseases via *de novo* insertion suggest that this subfamily is still actively retrotransposing [22,23]. The Yb8 subfamily is characterized by a tandem duplication of seven nucleotides from the 246th to the 252nd position of the AluY consensus sequence. The concurrent mutation and transposition of certain Yb8 elements generated the Yb9 subfamily, which was the latest Yb subfamily identified before this study and characterized by a C to G transversion at the 274th position [9]. In this study, using a computational approach we performed a genome-wide analysis of all human-specific Yb elements to identify their source copies and to track their recent evolutionary pathway. We successfully detected at least one driver copy for Yb8 and one Yb8 element that is potentially the source copy for the Yb9 subfamily. We also identified and characterized three new subfamilies in the Yb lineage: Yb8a1, Yb10, and Yb11. Yb11 is the youngest Yb subfamily reported to date.

## Results and discussion

### Evolution of recent *Alu* Yb elements

**Figure 1:**
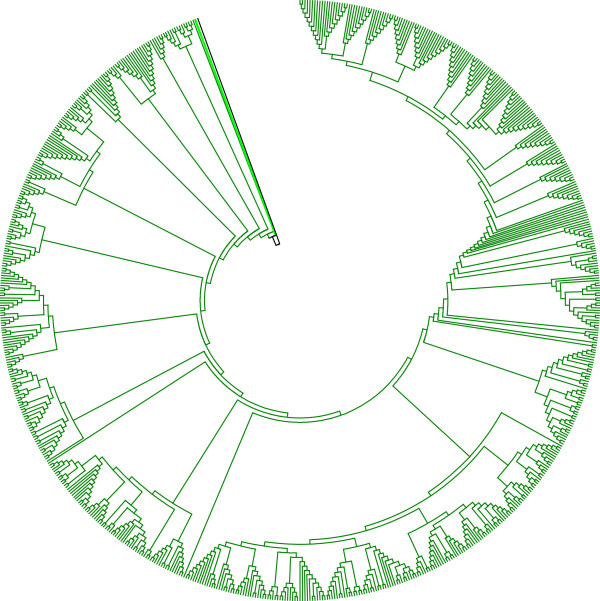
**Cladogram with 714 hsYb8 elements constructed by the neighbour joining method.** The element marked with a bold line (at hg19/chr10:10493416–10493732) is likely to be the source copy of all others in the tree. The tree was rooted using the Yb8 consensus, which is indicated by the black line.

Of all Yb copies found in the human genome, 80% (2,545 of 3,179) are identified as human-specific (hsYb), that is, they became integrated into the human genome after the human–chimp divergence, and they only include members of the Yb8 and Yb9 subfamilies (Tang *et al*., manuscript in preparation). In this study, we included all full-length hsYb elements in an attempt to assess their evolutionary pattern and backtrack their putative source genes. All such hsYb elements were aligned against all Yb7, 8 and 9 sequences in the reference genome to group similar sequences into clusters. For each cluster, a phylogenetic tree was constructed with an outgroup subfamily consensus sequence as its root to assess the evolutionary relation among clusters and members of each cluster. The phylogenetic topology for each cluster can provide information on the potential parent copy for other members in the cluster. In an analysis involving only hsYb8 elements and their best matches, one particular cluster consists of 714 Yb8 elements. The phylogenetic tree involving all of these elements indicates that one copy of Yb8 (at hg19/chr10:10493416–10493732) seemed to have generated multiple active Yb8 copies that further retrotransposed to produce eventually 713 copies or 54% of all 1,322 hsYb8 elements studied (Figure 1). This master Yb8 element was most likely the major driver of the Yb8 expansion after the human–chimp divergence. Eight other Yb8 elements were detected that generated at least ten copies of offspring Yb8 elements. These Yb8 elements with lower activity level comply with the 'stealth driver’ model of *Alu* evolution, which states that the stealth drivers do not generate as many copies of *Alu* as the master gene does, but rather function primarily to maintain the genomic retrotransposition capacity over a period of time [24].

**Figure 2:**
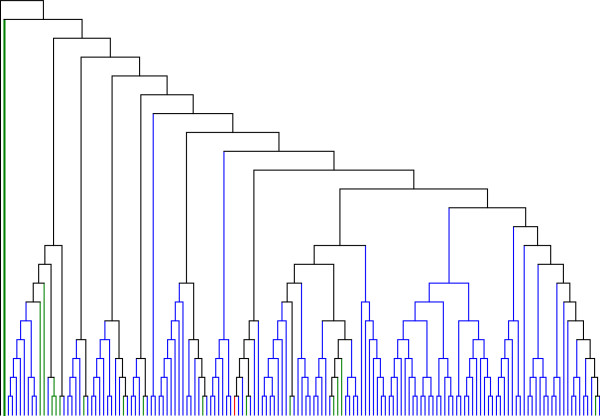
**Cladogram with 131 hsYb9 and 16 Yb8 elements constructed by the neighbour joining method.***Alu* Yb9 and Yb8 elements are shown in blue and green, respectively. There is one Yb8a1 element in the cluster that matches best with one of the Yb9 elements, shown in red. The Yb8 copy shown in bold green is likely to be the source of all Yb9 copies in the cladogram. The Yb8 consensus (root) is shown in black.

A similar approach was taken to track the evolutionary pathway of hsYb9 elements, involving identification and clustering of best-matched sequences from the whole genome. While almost all of the Yb9 elements tested aligned best with one another, 16 elements aligned best with 16 different Yb8 elements. When a phylogenetic tree was constructed with all hsYb9 elements and these 16 Yb8 elements, one particular Yb8 element at chr14:101990881–101991202 was found to be the source of all the hsYb9 elements, having generated multiple active Yb9 elements that subsequently generated 131 additional full-length hsYb9 copies (Figure 2). Along the evolutionary path of hsYb9, shown in Figure 2, some clusters have Yb8 elements, which may have resulted from either reverse mutation to produce Yb8 elements, or gene conversion or misannotation of Yb9 copies as Yb8 [25].

### Identification of novel *Alu* Yb subclasses

**Figure 3:**
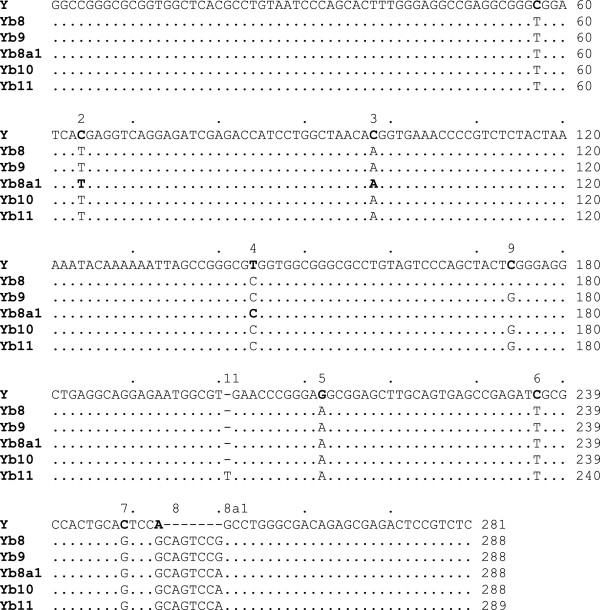
**Consensus sequences of *Alu *Y, Yb8, Yb9, Yb8a1, Yb10, and Yb11.** The signatory mutations are numbered in chronological order using Alu Y as the baseline.

Different subfamilies of the Yb lineage are characterized by specific mutations, and the subfamilies are defined according to the number of mutation sites with respect to the *Alu* Y consensus sequence [11]. Identification of new subfamilies is basically the identification of a set of *Alu* elements that share a particular mutation at a specific site that has not been previously reported. Using a computational approach, we performed a genome-wide analysis of *Alu* elements that are currently annotated as Yb8 and Yb9, the two most recent subfamilies of the Yb lineage known to date, to investigate whether any specific mutation beyond the Yb8 and Yb9 signature mutations is shared by more than one element. To do so, a set of full-length members of the *Alu* Yb8 and Yb9 subfamilies were retrieved from the latest assembly of the human reference genome sequence GRCh37, and multiple sequence alignment was performed after the poly-A segments were removed. Upon careful examination of the alignment data, two specific mutations were observed in multiple Yb9 and Yb8 elements at the 201st (insertion of T) and 259th (G → A) positions, respectively. We also observed that *Alu* sequences with the single base insertion after the 200th position always carry the mutation at the 259th position and the Yb9 diagnostic mutation at the 174th position, but not all sequences with a mutation at the 259th position contain the other two mutations. This is only possible if the sequences with the 259^G→A^ mutation originated from the Yb8 subfamily as the first event and then a subset of these sequences accumulated the Yb9-diagnostic 174^C→G^ mutation, or vice versa, giving rise to another new subfamily, which subsequently accumulated the 200^+T^ insertion to generate yet another subclass of Yb elements. Following the standard nomenclature of *Alu*s [11], we named the sequences with the 259^G→A^ mutation *Alu* Yb8a1, the sequences with the 259^G→A^ and 174^C→G^ mutations *Alu* Yb10, and the sequences with the 259^G→A^ and 174^C→G^ mutations and the 200^+T^ insertion *Alu* Yb11 (Figure 3)*.* When a Yb8a1 signatory sequence of 30 bases was constructed and aligned against the human reference genome, 99 Yb10 copies were identified, among which 75 copies did not have the 174^C→G^ mutation (Yb8a1), 8 had the 174^C→G^ mutation (Yb10), and 16 copies had both the 174^C→G^ mutation and the 200^+T^ insertion (Yb11). A 24-nucleotide-long signatory sequence was also constructed for Yb11, and when this sequence was aligned against the reference genome, 16 matches were detected, all of which overlap with the results from the Yb10 signatory sequence-whole genome alignment, which provides evidence for the accuracy of the method. In the end, we were able to detect 75 Yb8a1, 8 Yb10 and 16 Yb11 insertions in the reference genome (Additional file 1: Table S1).

Besides the reference genome, we also analysed 1000 Genome Project (1KGP) data and sequencing trace data from HuRef [26], to identify insertions of the newly identified subfamily members that are absent in the reference genome. We collected all of the Yb8 and Yb9 insertions that are absent from the reference genome but present in one or more individual genome sequences in the 1KGP data, for which sufficient insertion sequences could be constructed. Signature sequences for Yb8a1, Yb10 and Yb11 were then aligned against these sequences and the HuRef sequencing, resulting in the detection of an additional 6 Yb8a1, 3 Yb10 and 15 Yb11 insertions outside the reference genome. The insertion of T in the Yb11 elements outside the reference genome was confirmed by PCR amplification and sequencing for five of these 15 loci and by manually checking the sequencing data from the National Center for Biotechnology Information (NCBI) trace database for three of them (Additional file 2: Figures S1 and S2; Additional file 3: Table S2). Therefore, we were able to identify a total of 81 Yb8a1, 11 Yb10 and 31 Yb11 insertions, and we can expect that more of these will be identified after processing more personal genomes.

### Age estimation

Mutation densities were calculated for each subfamily to estimate the approximate age of the new subfamilies. Only full-length or near full-length *Alu* elements in the reference genome were considered (65 Yb8a1 out of 75, 8 Yb10, and 15 Yb11 out of 16) and the poly-A regions in the middle and at the end were removed. For the 65 elements from the Yb8a1 subfamily, the non-CpG mutation density was 0.29% (43 out of 14,625 total non-CpG bases). Using a neutral rate of evolution of 0.15% per million years for primate intervening DNA sequences [27] along with the non-CpG mutation density, the average age of the Yb8a1 subfamily was estimated to be 1.93 million years old. For the 8 Yb10 elements, 5 non-CpG mutations were detected out of a total of 1,904 non-CpG nucleotides constituting only 0.26% of them, indicating an estimated age of 1.73 million years for Yb10. For the Yb11 subfamily, 15 elements were analysed with a total of 3,720 non-CpG nucleotides; only 4 of these had mutated, yielding a neutral mutation density of 0.107% and an estimated age of 0.71 million years. To assess how recent these subfamilies are in relation to the already known Yb subfamilies, the age of Yb9 was also estimated. A total of 166 non-CpG mutations were identified from 254 *Alu* Yb9 family members containing 51,562 non-CpG nucleotides; 73 members were not included in the calculations due to a 5' truncation or a large deletion inside the Yb9 element. Using the same neutral rate of evolution and the non-CpG mutation density of 0.32% (166/51,562), the average age of the Yb9 subfamily members was estimated to be 2.15 million years. The age of the Yb9 subfamily estimated in this study is much older than that estimated initially by Roy-Engel *et al*. [9], mainly because the total number of Yb9 elements in their study was much smaller than in this study. However, our estimation of the age of Yb9 is very close to that identified in a similar study, which estimated the age of Yb9 as 2.32 million years [14]. The estimated age for Yb8a1 indicates that this subfamily originated almost at the same time as Yb9, providing evidence that Yb8a1 originated from Yb8. The Yb10 subfamily, which evolved 1.73 million years ago, should be mostly fixed across all human populations, while the Yb11 subfamily, at only 0.71 million years old, is most likely to be highly polymorphic among human populations because it is the youngest. The level of polymorphism for these newly identified subfamilies with respect to their ages are examined further in the following section.

### Level of polymorphism

The *Alu* Y family is evolutionarily the 'youngest’ *Alu* family and the Yb lineage was found to be one of the largest and most active lineages of all young *Alu* elements [12,14,28]. Out of the 2,433 full-length Yb elements found in the human genome, 499 were found to be polymorphic for their presence or absence between individuals and/or populations, and a further 304 Yb copies were identified in individual genome sequences that are not present in the reference genome [16,29]. Since the majority of Yb elements became inserted into the human genome 3 to 4 million years ago, we suspect that the very recently evolved subfamilies contribute most to the polymorphism due to the Yb lineage since the divergence of the various human populations from their common ancestor occurred only 100,000 years ago [14]. We assessed the level of polymorphism for all identified Yb8a1, Yb10 and Yb11 insertions by surveying *Alu* insertions and deletions in personal genomics data. We compared the insertions that are present in the reference genome with the structural variation data from the 1000 Genome Project [30]. Of these, 13 out of 16 (approximately 81%) Yb11 elements and 2 out of 8 (25%) Yb10 were found to be dimorphic, while 22 out of 75 (approximately 29%) Yb8a1 present in the reference genome are polymorphic. We then compared these polymorphic insertions with dbRIP to identify how many of them have previously been reported as polymorphic and found that 7 and 2 polymorphic Yb8a1 and Yb11 elements, respectively, overlap with dbRIP data [6]. Combining insertions both inside and outside the reference genome, a total of 28 out of 31 (approximately 90%) Yb11 and 5 out of 11 (approximately 45%) Yb10 were found to be polymorphic, while only 28 out of 81 (approximately 34%) of Yb8a1 insertions were identified as polymorphic. The difference in the level of polymorphism is inversely related to the age of the lineage, that is, the higher the polymorphism level among individuals and/or populations, the more evolutionarily recent the lineage. The difference in the fraction of polymorphic members among the three novel subfamilies confirms that Yb11 has evolved more recently than Yb10 and Yb8a1. The relative newness of the Yb11 lineage is further substantiated when we looked at the sequence divergence within the members of each subfamily (Table 1). The mean evolutionary divergence between each pair of sequences in the Yb8a1, Yb9, Yb10 and Yb11 subfamilies was estimated to be 0.016, 0.026, 0.015 and 0.006, respectively. The divergence value is directly related to the age of the population, that is, the older the set of sequences, the more evolutionarily divergent the sequences are. The mean divergence values provide another line of data suggesting that Yb8a1, Yb10 and Yb11 evolved chronologically during the evolution of humans.

**Table 1:** **Estimates of evolutionary divergence between and within full-length *Alu *** **Yb9, Yb10 and Yb11 elements**

	***Alu *** **Yb8a1**	***Alu *** **Yb9**	***Alu *** **Yb10**	***Alu *** **Yb11**
*Alu* Yb8a1	0.016^a^			
*Alu* Yb9	0.026^b^	0.026		
*Alu* Yb10	0.019	0.022	0.015	
*Alu* Yb11	0.015	0.017	0.011	0.006

^a^The average of base substitutions per site of all pairwise comparisons within the group.

^b^The average of base substitutions per site of all pairwise comparisons among the members of the two groups compared.

**Figure 4:**
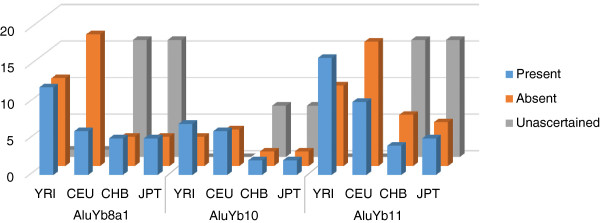
**The level of polymorphisms for the Yb8a1, Yb10 and Yb11 subfamilies.** The blue columns at the front indicate the number of polymorphic insertions observed in the population and the orange columns in the middle represent the number of insertions observed in other populations but not in the population. The presence or absence of polymorphic insertions in Chinese and Japanese populations could not be determined and these are labeled as 'unascertained’ and represented by grey bars. CEU, Utah residents with European ancestry; CHB, Han Chinese from Beijing areas; JPT, Japanese from the Tokyo area; YRI, Yoruban.

We also examined the distribution of all polymorphic members of Yb8a1, Yb10 and Yb11 in Yoruban, European, Chinese and Japanese populations. It was observed that 50%, 64% and 59% of polymorphic elements are present in the Yoruban population for the Yb8a1, Yb10 and Yb11 subfamilies, respectively (Figure 4). These numbers are higher than the equivalent numbers for the other non-African populations examined. The highest number of polymorphic elements were expected to be present in the Yoruban population as this was the oldest population tested in this study [31]. While the presence or absence of some of the polymorphic elements could not be ascertained for the Chinese and Japanese populations (they are flagged as 'unascertained’), the majority of the rest (approximately 66%) were present in one or both of the Asian populations. Among these, only one Yb8a1 insertion was found to be specific to the Chinese population and the rest are all shared by one or more other populations. In contrast, 15 Yb8a1, 5 Yb10 and 10 Yb11 insertions are specific to the Yoruban population, and 2, 3 and 4 of each of Yb8a1, Yb10 and Yb11 insertions are specific to the European population. This suggests that the number of population-specific insertions decreases with the age of the population. In other words, the older the population, the more time there has been for active young *Alu* elements to retrotranspose, creating a direct relation between the number of population-specific *Alu*s and the age of population.

### Evolutionary pathways for the three new *Alu* Yb subfamilies

New *Alu* families are created when a mutation occurs in the master or source active *Alu* element, which subsequently retrotransposes to give rise to a new lineage of *Alu* elements that share the same diagnostic mutation. The master gene model is the most widely accepted model for the generation of new *Alu* subfamilies [8] even though there many doubts about the details of this model [10,32-34]. While this model only gives a hierarchical evolution for the different subfamilies, the specific evolutionary pathways for the generation of different Yb lineages have yet to be characterized. The evolution of Yb9, Yb8 and Yb7, the three most recent and abundant subfamilies of the Yb lineage, occurred sequentially [9].

**Figure 5:**
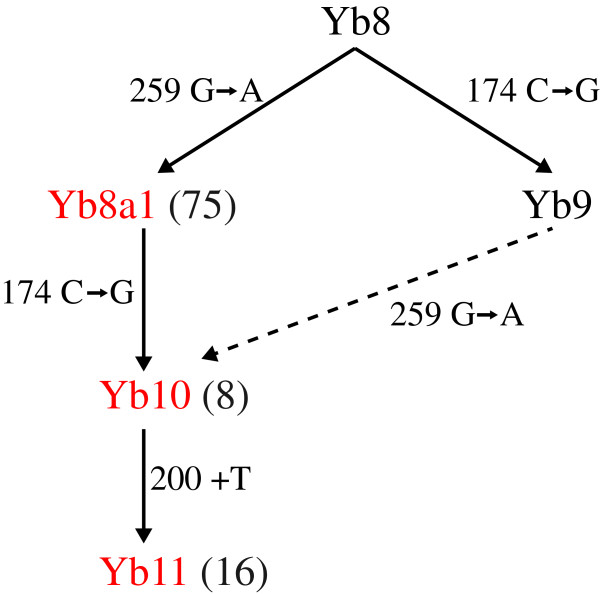
**Evolution of the recent *Alu *Yb lineage.** The subfamilies in black are the current known subfamilies and the subfamilies in red are novel and proposed in this study. The numbers accompanying each subfamily are the total number of copies found in the human reference genome. The dotted line is the less convincing alternative pathway for the evolution of the Yb10 subfamily.

**Figure 6:**
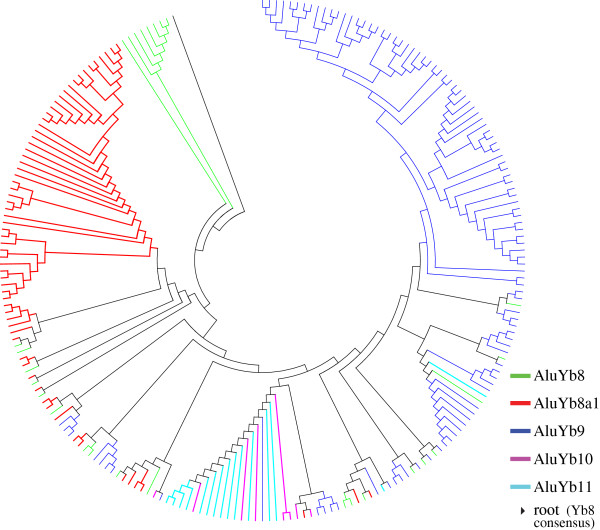
**Cladogram of all full-length Yb9, Yb8a1, Yb10, and Yb11 elements using the neighbour joining method.** The tree is rooted with the *Alu* Yb8 consensus sequence, which is shown in black at the top left.

In our study, we predict that the evolution of Yb11 took a strict sequential linear pathway from Yb10 since it contains one more mutation than Yb10 diagnostic mutations, while the Yb10 subfamily evolved from either Yb8a1 or Yb9 following one or more pathways (Figure 5). A tree using the neighbour joining method was constructed among 25, 181, 65, 8 and 15 full-length Yb8, Yb9, Yb8a1, Yb10 and Yb11 elements, respectively, rooted with the Yb8 consensus sequence (Figure 6). The 25 Yb8 elements were included because these are the only Yb8 copies that one or more of Yb9, Yb8a1, Yb10 and Yb11 had the best similarity score with. It was observed from the topology that 77% of all Yb8a1 elements have evolved from one individual Yb8 copy, and 63 out of 65 Yb8a1 copies tested are evolutionarily closest to members of the Yb8 subfamily. This confirms that Yb8a1 evolved from Yb8 as a separate lineage from Yb9. Among the 15 Yb11 copies included in the phylogenetic analysis, all of them have common nodes with copies from Yb10 elements, confirming their linear evolutionary pathway from the Yb10 subfamily.

The diagnostic mutations of the Yb10 subfamily are predicted to have evolved by following one of two pathways: (1) a Yb9 element obtained the Yb8a1-specific mutation and retrotransposed to generate the Yb10 subfamily or (2) a Yb8a1 element obtained the Yb9-specific mutation subsequently generating the Yb10 subfamily. The phylogenetic analysis on its own does seem to favour the latter option since the major branch leading to the Yb10/Yb11 lineage is closer to the Yb8a1 cluster. For additional evidence, an evolution network was constructed for all full-length members of the four subfamilies of interest using the median joining method [35]. The network shows that the majority of the Yb10 elements are linked closer to multiple Yb8a1 elements than to Yb9 (Additional file 2: Figure S3), further supporting the prediction that the evolution of Yb10 was from Yb8a1 by gaining the Yb9 mutation. The accumulation of the Yb9-specific mutation in the Yb8a1 copy parent to create the Yb10 subfamily may have occurred by gene conversion and requires further analysis for confirmation. A second line of evidence for the evolutionary pathway proposed here is provided by the linear pairwise evolutionary distances calculated for the Yb9, Yb8a1, Yb10 and Yb11 elements (Table 1). The mean evolutionary distance for all sequences between Yb10 and Yb11 was calculated as 0.011, which is lower than the distance between Yb9 and Yb11 (0.017) or Yb8a1 and Yb11 (0.015) indicating the sequential evolution of Yb11 from Yb10 and with Yb8a1 being closer than Yb9 to Yb11.

Each of the Yb8a1, Yb10 and Yb11 subfamilies was also tested using the molecular clock (ML) to assess if all full-length members in each subfamily evolved at a homogeneous rate. A maximum likelihood test of the ML hypothesis was performed separately for each of the Yb8a1, Yb10 and Yb11 phylogenetic tree topologies and sequence alignments [36]. The ML hypothesis states that all tips of the tree should be equidistant from the root of the tree, or in other words the rate of evolution of all branches in the tree is uniform. The maximum likelihood, *–*ln *L*, was calculated to be 990.971 and 907.158 for with-clock and without-clock phylogeny, respectively, for Yb8a1, 466.906 and 455.855 for with-clock and without-clock phylogeny, respectively, for Yb10, and 481.574 and 474.459 for with-clock and without-clock phylogeny, respectively, for Yb11. The chi-square test based on the difference in the likelihood ratio between with-clock and without-clock phylogeny rejected the null hypothesis of uniform evolution for both *Alu* Yb8a1 and Yb10 insertions at a 5% significance level with *P* < 0.0001 and *P* < 0.001 for Yb8a1 and Yb10, respectively. However, we failed to reject the null hypothesis of an equal evolutionary rate for all Yb11 insertions at a 5% significance level (*P <* 0.43)*.* This indicates that neither the Yb8a1 nor the Yb10 subfamily evolved at a uniform evolutionary rate, and that the evolution of the subfamily Yb11 has been uniform. This provides further evidence that the Yb8a1 and Yb10 subfamilies are older than the Yb11 subfamily since evolutionary uniformity is more likely in a recently evolved lineage. Furthermore, when the evolutionary relations for all full-length Yb8a1, Yb9, Yb10 and Yb11 elements were analysed, more divergence among members of Yb8a1 and Yb9 was observed than among the members of Yb10 or Yb11 (Additional file 2: Figure S4), another indication that the former subfamilies are older than the latter.

## Conclusions

The *Alu* Yb lineage has an extended evolutionary history in the human genome. Even though the lineage evolved before the human–chimp divergence, most of the insertions occurred in the last 3 to 4 million years and some copies of this lineage still retain the ability to retrotranspose. One such active Yb8 copy has generated almost 60% of all human-specific Yb8 copies and several others have generated more than ten copies, indicating the presence of both a master copy and stealth drivers for this subset of Yb8 elements.

The tracking of the source copy in this study enabled us to identify the potential master gene of all Yb9 elements. The relatively higher activity of the Yb lineage than almost all other *Alu* lineages has generated several subfamilies that were previously undetected and which share a specific pattern of mutations. Three such novel subfamilies proposed in this study are Yb8a1, Yb10 and Yb11. Even though Yb8a1 and Yb10 are believed to have evolved within a short time of each other, only eight copies of Yb10 have been detected in the human reference genome compared to 75 copies of Yb8a1. Furthermore, Yb9 has been estimated to be only 0.22 million years older than Yb8a1, yet the number of Yb9 copies in the human genome is almost five times larger than the number of Yb8a1 copies. This indicates that not all of the *Alu* subfamilies grew at an equal rate and that some mutation patterns may accelerate the rate of transposition. This is further supported by the fact that the Yb11-specific insertional mutation in the Yb10 sequence has accelerated the rate of retrotransposition resulting in 16 copies of Yb11 since it first evolved 0.71 million years ago. The possibility that certain mutations accelerate the rate of transposition and their mechanism should be the subject of further study.

Yb11 is the latest subfamily to have evolved in this lineage and it is highly polymorphic among different individuals and/or populations. The generation of these young subfamilies indicates that *Alu*s are still evolving, and this provides some clues regarding the future trend of *Alu* activity in the human genome. The homoplasy-free nature of *Alu* insertions makes these very recent genetic variants a valuable resource in forensics and for studying modern human population genetics and migration patterns.

## Methods

### Source copy tracking

All human-specific Yb elements were retrieved from a separate study (Tang *et al*., unpublished data). The human-specific Yb lineage has members from only Yb8, Yb9 and the newly identified subfamilies. Each full-length human-specific Yb element was aligned against the reference genome using BLAST [37] with the e-value set to 10^-5^. Based on the BLAST results, any insertions that match more than one genomic region with equal matching quality were omitted from further analysis as the source copy of these insertions could not be determined. The remaining sequences were divided into clusters based on their similarity with one another. The evolutionary relation between members of each cluster was obtained by constructing a phylogenetic tree using the neighbour joining method rooted with the Yb8 consensus sequence, and some cases were supplemented with network analysis using the median joining method [35].

### Identification of new *Alu* Yb subfamilies

Position information for all *Alu* Yb8 and Yb9 elements from the latest major version of the human genome assembly GRCh37 were retrieved from the RepeatMasker track of the UCSC genome browser [38] and the sequence for each insertion was retrieved from the reference genome. The poly-A segments from both the 3' end and the middle were removed manually. The pairwise alignment for all Yb9 sequences was visualized in MEGA5 [39]. A signatory sequence was constructed encompassing each of the signature insertions at the 201st position and the mutation at the 259th position. The sequences were conserved across all *Alu* Yb insertions except for the mutation/insertion base. These sequences were aligned against the reference genome using BLAST with an e-value of 10^-5^. The resulting matches were filtered using an in-house Perl script to retain only the sequences that have the signature mutation/insertion. To identify additional insertions of the new subfamilies that are absent in the reference genome, genome sequencing and alignment data from the 1000 Genome Project were downloaded to our local server. New insertions for *Alu* Yb8 and Yb9 in the six high coverage genome datasets from phase 1 of the 1000 Genome Project were identified in a separate study [40]; the read cluster for each predicted novel insertion contains all reads from the inserted region. From the mobile element insertion list generated from the pilot phase 1 data of the 1000 Genome Project [16], we collected 304 *Alu* Yb8 and Yb9 insertions that are absent in the reference genome but were detected in one or more of the test genomes for which a complete insertion sequence could be constructed. A custom BLAST database was created to contain all these new insertion sequences, and the signature sequences were aligned against this custom database using the abovementioned criteria.

### Validation of Yb11 insertions outside the reference genome

The insertion of T after the 200th nucleotide in Yb11 can potentially be the result of a sequencing error since the preceding base is also a T. To eliminate the possibility of erroneous results, all reads sequenced by Sanger’s method were downloaded from the NCBI trace database to our local server. The Yb11 signatory sequence was aligned against these reads to identify the reads that contain Yb11. A total of 130 reads were found to contain the Yb11-specific T insertion. The Phred quality score of the site of the T insertion in each read was analysed using a custom Perl script (Additional file 2: Figure S1). Three out of fifteen loci could be confirmed using these trace data. Of the remaining twelve Yb11 insertions that are outside the reference genome sequence, primers could be designed for six *Alu* insertions. Five insertions could be amplified by PCR in DNA samples NA19239 and NA19240 from the Coriell Cell Repositories [41] and an in-house mixed DNA, all of which received approval from the Brock University Research Ethic Board. The amplified products were sequenced using the Sanger method at The Centre for Applied Genomics. The sequencing primers include locus-specific flanking primers and two *Alu*-internal primers designed from the 5' and 3' ends of the Yb11 consensus sequence, which are TGGCTCACGCCTGTAATC and GACGGAGTCTCGCTCTGTC, respectively. The internal primers help with difficulties in sequencing through the poly-A regions within *Alu* sequences. The sequences were aligned using clustalW to analyse the Yb11-specific site (Additional file 2: Figure S1). All new *Alu* insertion sequences not covered by dbRIP were processed for deposition into dbRIP [42] under the study ID 2013–02.

### Analyses of the Yb8a1, Yb10 and Yb11 insertion polymorphisms and evolution relations

To assess the level of polymorphism among the insertions of the three new subfamilies, the start and end position of each insertion was compared with structural variation [30] and mobile element insertion [16] data from the 1000 Genome Project and with entries from dbRIP [6]. The phylogenetic tree for all full-length *Alu* Yb9, Yb8a1, Yb10 and Yb11 insertions along with the putative source Yb8 copies obtained from previously mentioned clusters was constructed using the neighbour joining method [43]. All alignments and phylogenetic trees were visualized using the MEGA software [39]. The evolutionary distance and sequence divergence within and between subfamilies were calculated using the maximum composite likelihood model [44] involving 181 full-length Yb9, 65 Yb8a1, 8 Yb10 and 15 Yb11 nucleotide sequences without poly-A sequences at the 3' end and in the middle.

## Abbreviations

1KGP: 1000 genome project; hsYb: Human-specific *Alu* Yb; ML: Molecular clock; NCBI: National center for biotechnology information; PCR: Polymerase chain reaction; SINE: Short interspersed element.

## Competing interests

The authors declare that they have no competing interests.

## Authors’ contributions

MA collected all data, carried out the analysis and drafted the manuscript. WL performed the PCR analysis and sequencing. PL conceived the study, supervised the design and all analyses performed, and also edited the manuscript to its final version. All authors read and approved the final manuscript for publishing.

## Supplementary Material

Additional file 1: Table S1 List of Yb8a1, Yb10 and Yb11 insertions identified in the reference genome. Click here for file

Additional file 2: Figure S1 to S4 Contains Figure S1 to S4 to supplement the PCR and evolutionary analysis data presented in the article. Click here for file

Additional file 3: Table S2 List of Yb8a1, Yb10 and Yb11 insertions identified outside the reference genome. Click here for file
