# Supplementary material for: Identification of three new Alu Yb subfamilies by source tracking of recently integrated Alu Yb elements
Source: Mob DNA. 2013 Nov 12;4:25. doi: 10.1186/1759-8753-4-25 (PMC3831846; doi:10.1186/1759-8753-4-25)
Supplement: Additional file 2: Figure S1 to S4 — Contains Figure S1 to S4 to supplement the PCR and evolutionary analysis data presented in the article. [file 1759-8753-4-25-S2.docx]

a)
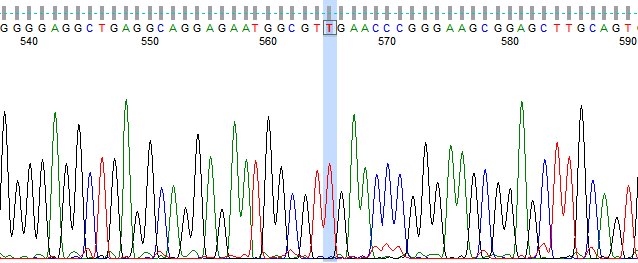


b)

**Supplementary Figure 1: Identification and quality analysis of Yb11-specific insertion of T in human genome fragments sequenced by Sanger’s method obtained from NCBI database.** a) Chromatograph of a sequence read output from Sanger’s method (TI: 1747216562). The Yb11-specific insertion of T is highlighted. The top bars above the nucleotide labels represent the Phred quality scores for individual bases. b) The Phred quality score of Yb11-specific insertion site in all reads that have the Yb11 sequence. Each bar represents the site of T-insertion in each individual sequence read. A Phred score of 10 denotes 90% base call accuracy, 20 denotes 99% accuracy and 50 denotes 99.999% accuracy. A Phred score of 0 indicates that no base call could be made due to poor sequencing quality.

3528_predicted AATTAGCCGGGCGTGGTGGCGGGCGCCTGTAGTCCCAGCTACTGGGGAGGCTGAGGCAGG 60

108507 AATTAGCCGGGCGCGGTGGCGGGCGCCTGTAGTCCCAGCTACTGGGGAGGCTGAGGCAGG 60

3528 AATTAGCCGGGCGCGGTGGCGGGCGCCTGTAGTCCCAGCTACTGGGGAGGCTGAGGCAGG 60

128385 AATTAGCCGGGCGCGGTGGCGGGCGCCTGTAGTCCCAGCTACTGGGGAGGYTGAGGCAGG 60

56065 AATTAGCCGGGCGCGGTGGCGGGCGCCTGTAGTCCCAGCTACTGGGGAGGCTGAGGCAGG 60

55925 AATTAGCCGGGCGCGGTGGCGGGCGCCTGTAGTCCCAGCTACTGGGGAGGCTGAGGCAGG 60

************* ************************************ *********

3528_predicted AGAATGGCGT**T**GAACCCGGGAGGCGGAGCTTGCA 94

108507 AGAATGGCGT**T**GAACCCGGGAAGCGGAGCTTGCA 94

3528 AGAATGGCGT**T**GAACCCGGGAAGCGGAGCTTGCA 94

128385 AGAATGGCGT**T**GAACCCGGGAAGCGGAGCTTGCA 94

56065 AGAATGGCGT**T**GAACCCGGGAAGCGGAGCTWGCA 94

55925 AGAATGGCGT**T**GAACCCGGGAAGCGGAGCTTGCA 94

********************* ******** ***

**Supplementary Figure 2: Alignment of the partial sequences of amplified Yb11 loci that are absent in the reference genome but present in one or more other individual genome sequences.** Sequencing was done using Sanger’s method and all bases used in this alignment were called with Phred quality score of above 20. The Yb11-specific insertion of T is highlighted. The amplified loci are with IDs of 55925, 3528, 128385, 56065 and 108507. “3528_predicted” is the predicted sequence obtained from the 1000 Genome Project data for the locus ID P1_MEI_3528&P2_MEI_466.

**Supplementary Figure 3: Network between full length *Alu* Yb8, Yb9, Yb8a1, Yb10 and Yb11 elements using Median Joining method.** Each subfamily forms a cluster based on sequences that is annotated by circles. The length of each connecting line is relative to the number of mutations. The novel *Alu* subfamily Yb8a1 is closer to Yb8 cluster, Yb10 is closer to Yb8a1 and Yb11 has connection only with Yb10 members.

**Supplementary Figure 4: Evolutionary relationships of all full-length Yb9, Yb8a1, Yb10 and Yb11 elements.** The green, blue, red, magenta and neon lines represent Yb8, Yb9, Yb8a1, Yb10 and Yb11 elements respectively. The tree is rooted with AluYb8 consensus sequence. The evolutionary history was inferred using the Neighbor-Joining method. The tree is drawn to scale, with branch lengths in the same units as those of the evolutionary distances used to infer the phylogenetic tree. The evolutionary distances were computed using the Maximum Composite Likelihood method and are in the units of the number of base substitutions per site. All ambiguous positions were removed for each sequence pair. There were a total of 254 positions in the final dataset.
